# Seldinger Technique for Placement of “Peripheral” Internal Jugular Line: Novel Approach for Emergent Vascular Access

**DOI:** 10.5811/westjem.2015.11.28726

**Published:** 2016-01-12

**Authors:** Adam J. Ash, Christopher Raio

**Affiliations:** * North Shore University Hospital, Department of Emergency Medicine, Manhasset, New York; † Good Samaritan Hospital Medical Center, Department of Emergency Medicine, West Islip, New York

## Abstract

This is a case report describing the ultrasound-guided placement of a peripheral intravenous catheter into the internal jugular vein of a patient with difficult vascular access. Although this technique has been described in the past, this case is novel in that the Seldinger technique was used to place the catheter. This allows for safer placement of a longer catheter (2.25″) without the need for venous dilation, which is potentially hazardous.

## INTRODUCTION

Peripheral intravenous (IV) access is an essential component of emergency department (ED) care. Traditionally, success rates are high.^1^,^2^ There are, however, subsets of patients, including the morbidly obese, chronically ill, severely dehydrated, or those who use intravenous drugs, in whom peripheral venous access is difficult or impossible to obtain using conventional methods.^3^ These patients represent a unique challenge to emergency and critical care physicians, and multiple alternative strategies have been described to assist in this setting. These include placement of a catheter in an external jugular (EJ) vein, blind placement into a deep (brachial) upper arm vein, ultrasound-guided placement in a peripheral vein, or placement of central venous catheter. In critically-ill patients, an intraosseous catheter or even saphenous vein cutdown may also be used.^4^

A novel technique that has been described is the placement of a standard IV catheter into the internal jugular vein under dynamic ultrasound-guidance.^4^,^5^ Although limited data are available, there have been no reported complications secondary to the use of this technique.

**Figure 1:**
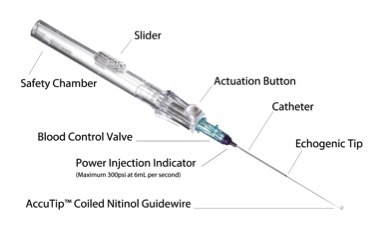
AccuCath® 2.25″ BC catheter.

At our institution, we perform ultrasound-guided placement with the AccuCath^®^ 2.25″ BC intravascular catheter (Bard Access Systems, Salt Lake City, UT) to obtain vascular access in challenging patients (Figure 1). This is an intravascular catheter that integrates a coiled tip nitinol guidewire, which minimizes the need for needle advancement. The guidewire technology aids in navigating vessel anatomy to achieve atraumatic delivery of the catheter using the Seldinger technique. Here, we describe a novel technique for obtaining vascular access through the internal jugular vein using ultrasound-guided placement of this device.

## CASE REPORT

A 69-year-old female with a history of diabetes mellitus, hypertension, and end-stage renal disease presented to our ED via ambulance after becoming hypotensive during hemodialysis. She complained of mild fatigue and weakness, but had no other complaints and stated that this had happened to her previously, and that she typically improved with intravenous (IV) hydration. She also stated that, because she has had multiple arteriovenous shunts in the upper extremities, obtaining peripheral IV access was typically not possible and that she usually required a central venous catheter. On physical examination, her blood pressure was 89/45, heart rate 92 beats per minute, respiratory rate 12 breaths per minute, temperature 37.1 degrees Celcius with a room air oxygen saturation of 98%. The patient was completely alert and oriented and in no acute distress, but appeared to be mildly fatigued. She had a right internal jugular hemodialysis catheter and scarring on the skin of her upper extremities noted in the vicinity of prior vascular access sites. The remainder of her examination was unremarkable.

**Figure 2:**
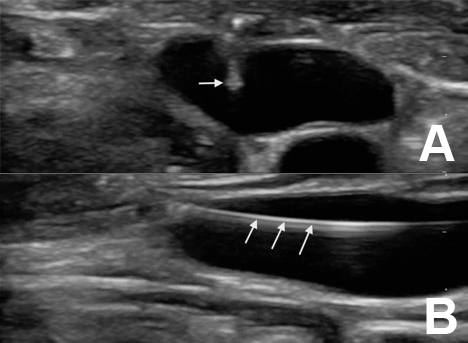
A) Cross-sectional view of the left internal jugular vein with needle in lumen. B) Longitudinal view of guidewire in lumen of left internal jugular vein.

Initial attempts to establish peripheral vascular access with and without ultrasound guidance were unsuccessful. However, given the patient’s relatively well appearance, and the desire to avoid dilation of the left internal jugular vein (one of the patients last viable dialysis access sites), we opted to place an AccuCath^®^ 2.25″ BC catheter in this central vein under ultrasound guidance. The neck was prepped in sterile fashion and the left internal jugular vein was cannulated under dynamic ultrasound guidance using a Zonare Z.One PRO ultrasound system (Mountain View, California) (Figure 2a). The guidewire was then gently advanced into the vein (Figure 2b), and the catheter was subsequently advanced over the wire. One liter of IV normal saline was infused after which the patient’s blood pressure improved. She felt much better, and was discharged home after a brief observation period in the ED.

## DISCUSSION

Since its introduction in 1953, the Seldinger technique has been used with great success to cannulate a multitude of blood vessels, hollow viscous structures, and potential anatomical spaces. Prior to its introduction, central vascular access required the use of large bore needles through which smaller catheters were placed. This limited cannulation to larger blood vessels, and due to both the large needle and greater vessel sizes increased the risk of vessel damage and hemorrhage.^6^ Seldinger’s technique of placing a guide wire through the needle allowed for a catheter the same bore as the needle to be placed, enabling the placement of smaller catheters into smaller vessels, and decreasing the associated complications.^6^

The addition of ultrasound has been shown to decrease complications even further.^7^ In spite of this, however, accidental arterial puncture and dilation remain problematic.^8^ This is because even under ultrasound guidance, the needle can temporarily slip out of the visualized field and penetrate deeper than the operator desires.^9^ Arterial puncture is generally well tolerated and can be treated conservatively in most cases. Dilation, however, can lead to significant morbidity and mortality in the form of arterial dissection, hemorrhage, and arteriovenous fistula formation.^8^

Placement of a 1.88″ (48mm) angiocatheter into the internal jugular vein has been described in the literature,^4^,^5^ and is one technique that can be used to avoid accidental arterial puncture and dilation in difficult cases. The addition of the AccuCath^®^ 2.25″ BC catheter to this method allows clinicians to benefit from the advantages of the Seldinger technique without the risks of arterial cannulation and dilation. It can be performed more quickly than central venous access via traditional methods^4^ and, because the guidewire is much shorter than those typically used for central venous catheterization, the incidence of guidewire-induced cardiac dysrhythmias should be much lower.^10^ The integrated guidewire technology also helps to prevent vascular trauma and posterior wall damage.^6^,^8^ Although its use for central placement requires further study, it should be considered in difficult vascular access patients who do not require large bore or multi-lumen catheters.
